# Justification for species selection for pharmaceutical toxicity studies

**DOI:** 10.1093/toxres/tfaa081

**Published:** 2020-11-24

**Authors:** Helen Prior, Richard Haworth, Briony Labram, Ruth Roberts, Alison Wolfreys, Fiona Sewell

**Affiliations:** National Centre for the Replacement, Refinement and Reduction of Animals in Research (NC3Rs), 215 Euston Rd, London, NW1 2BE, UK; GlaxoSmithKline R&D, Park Road, Ware, SG12 0DP, UK; National Centre for the Replacement, Refinement and Reduction of Animals in Research (NC3Rs), 215 Euston Rd, London, NW1 2BE, UK; ApconiX, Alderley Park, Alderley Edge, SK10 4TG, UK; UCB Biopharma, Bath Rd, Slough, SL1 3WE, UK; National Centre for the Replacement, Refinement and Reduction of Animals in Research (NC3Rs), 215 Euston Rd, London, NW1 2BE, UK

**Keywords:** 3Rs, dog, drug development, minipig, non-rodent, rat, rodent, safety assessment, toxicology

## Abstract

Toxicity studies using mammalian species are generally required to provide safety data to support clinical development and licencing registration for potential new pharmaceuticals. International regulatory guidelines outline recommendations for the order (rodent and/or non-rodent) and number of species, retaining flexibility for development of a diverse range of drug modalities in a manner relevant for each specific new medicine. Selection of the appropriate toxicology species involves consideration of scientific, ethical and practical factors, with individual companies likely having different perspectives and preferences regarding weighting of various aspects dependent upon molecule characteristics and previous experience of specific targets or molecule classes. This article summarizes presentations from a symposium at the 2019 Annual Congress of the British Toxicology Society on the topic of species selection for pharmaceutical toxicity studies. This symposium included an overview of results from a National Centre for the Replacement, Refinement and Reduction of Animals in Research (NC3Rs) and Association of British Pharmaceutical Industry (ABPI) international collaboration that reviewed the use of one or two species in regulatory toxicology studies and justification for the species selected within each programme. Perspectives from two pharmaceutical companies described their processes for species selection for evaluation of biologics, and justification for selection of the minipig as a toxicological species for small molecules. This article summarizes discussions on the scientific justification and other considerations taken into account to ensure the most appropriate animal species are used for toxicity studies to meet regulatory requirements and to provide the most value for informing project decisions.

## Introduction

Toxicity assessments in animals form an integral part of the drug development process, providing data to support the design of human clinical trials and the safety of participating volunteers and patients. Studies are conducted in accordance with international regulatory guidelines (The International Council for Harmonisation of Technical Requirements for Pharmaceuticals for Human Use [1]) that describe high-level recommendations for the design and conduct of a range of studies (general toxicology, safety pharmacology, genetic toxicology, developmental and reproductive toxicology, carcinogenicity) to permit assessment of risk in relation to the potential benefit of the new medicine. The guidelines are intended to be flexible, so that the most appropriate approach can be taken for an individual product, including the use of *in vitro* and *in silico* methods where possible. Whilst the use of alternative approaches is increasing for screening purposes and for specific tests within the fields of absorption, distribution, metabolism and excretion (ADME), safety pharmacology and genotoxicity [2], many components of regulatory general toxicology assessments rely on data from animals and are likely to do so for the foreseeable future, until reliable alternatives are available and widely accepted as replacements for animal use. Until this time, opportunities to apply the 3Rs within toxicology studies include refinements within study designs to benefit animal welfare and study data [3–5] and reduction in animals by optimizing group sizes and study designs [6–8].

For small molecule new chemical entities following the ICHM3 (R2) guideline [9], studies using two species—a rodent and non-rodent—are generally expected; this is also the case for oncology products following ICHS9 [10]*.* The most common species tend to be rat and dog [11, 12], although non-human primates (NHPs) are also widely used as the non-rodent [13]*.* For biotechnology products such as monoclonal antibodies (mAbs) following the ICHS6 (R1) guideline [14], only studies in pharmacologically relevant species are expected, with studies in non-relevant species actively discouraged to prevent misleading results. Pharmacological relevance is generally demonstrated by a species that expresses the target antigen and evokes a similar pharmacological response as that expected in humans. As many biotherapeutic products are highly selective, often there is only one relevant species (frequently the NHP, owing to higher genome sequence homology to humans and similarity in physiological systems, such as the immune system) and single species programmes in NHP are common. However, if a rodent species is also pharmacologically relevant, toxicity studies in two species are recommended and this appears to be the case for 30–40% of mAbs [15–17].

The selection of the most appropriate species to use for toxicology studies is an important consideration, which is taken early within drug discovery based on scientific, ethical and practical factors. These include comparisons (similarities and differences) between various species and humans for target receptor expression, homology, distribution and subtypes, metabolic profile, pharmacokinetic (PK) profile, plasma protein binding and whether the pharmacology and physiology represent the expected effect in humans, to demonstrate relevance of the animal model [18]. Other considerations include knowledge/experience from previous or similar molecules regarding potential for tolerability issues which may limit the ability to achieve study objectives, such as histamine release [19] or emesis [20], adequate historical background data for different species and strains, and practical aspects such as ease of running studies using certain routes of administration or inclusion of specific procedures. Some individual companies or wider geographical regions (such as the EU) place particular emphasis on minimizing the use of certain non-rodent species for ethical reasons—preferring the use of minipig over dog [21, 22] or vice versa, or only permitting use of NHPs when other species are proved unsuitable [23, 24].

The number of species (and strains) considered and tested for suitability within each project is also dependent on company policy and practice, tailored to the class of molecule being developed. Although species choice is not prescribed within regulatory guidelines, only a few species are commonly used: mouse, rat, rabbit, dog, minipig or NHP. Data from these species have contributed to safe administration in humans for the majority of drugs in development, with reasonable predictivity for likely adverse events in those molecules that achieved entry into the clinic [13, 25, 26]. The purpose of this manuscript is not to debate the topic of predictivity or whether any of the toxicology species may be more useful than another [27, 28], but to provide examples for the scientific justification of the species chosen, when toxicology studies are required.

A symposium was held in April 2019 as part of the British Toxicology Society (BTS) Annual congress (Cambridge, UK). The speakers and audience explored outcomes of a large data-sharing project and case studies from two major UK pharmaceutical companies, to consider the justification for appropriate species choice for toxicology studies. The individual presentations and resulting discussions are summarized herein.

## NC3Rs/ABPI review of two species use: justification for species selection (*Helen Prior, NC3Rs*)

A recent collaboration between the NC3Rs and the ABPI aimed to review the species used for general toxicology studies of drug candidates within current pre-market portfolios [29]. Whilst the main focus was to explore opportunities for the use of a single species to support safe progression in humans, questions were also included within the data gathering survey to investigate the justification for the choice of each nonclinical species used for toxicity testing for the different molecule types within the cross-industry dataset (see Figs 2–4 notes for details).

**Figure 1:**
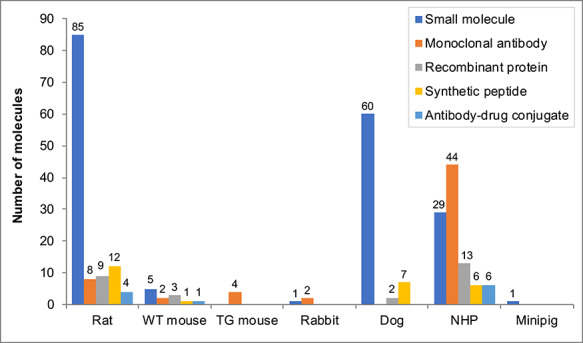
Species used for different molecule types within the NC3Rs/ABPI ‘Two species’ working group dataset. Note: The species used for general toxicology studies up to the current stage of drug discovery or development (excluding safety pharmacology, reproductive and developmental toxicology and carcinogenicity studies). Molecules had reached different stages of development, as outlined previously [17]. WT mouse is a wild-type strain of mouse; TG mouse is a transgenic mouse model. The NHP was cynomolgus monkey for all but three molecules; the rhesus macaque was used for one small molecule and two recombinant proteins. The numbers within the chart gives the actual number of molecules, for ease of reading.

The international working group reviewed anonymized data for 172 drug candidates received from 18 different organizations, consisting of 92 small molecules, 46 mAbs, 15 recombinant proteins, 13 synthetic peptides and 6 antibody-drug conjugates (ADCs). Toxicology studies were conducted in both a rodent and non-rodent species for the majority of small molecules, recombinant proteins, synthetic peptides and ADCs (97%, 80%, 100% and 83% of each drug modality, respectively), whereas a large number of mAbs (65%) were tested in a single non-rodent species [17]. The species used for toxicity testing of the small molecules in the dataset were predominantly rat, dog and NHP (Fig. 1). The NHP was also used for all ADCs, for the majority of mAbs (96%) and recombinant proteins (87%) and half of the synthetic peptides (the dog being the non-rodent species used for these latter drug candidates). The rat was also used for testing of 17% mAbs, 60% recombinant proteins, 92% of synthetic peptides and 66% ADCs. The mouse (both wild-type and transgenic models) or rabbit were also used for testing a small number of biologicals within the dataset (Fig. 1).

**Figure 2:**
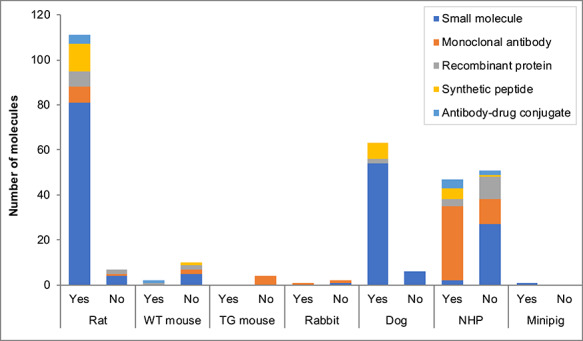
Was the species choice a standard company practice? Note: The *x*-axis shows the species used within toxicology studies and answers to the follow-up question ‘Was the species choice a standard company practice?’. Positive (Yes) responses reflect a standard company practice, defined as ‘the normal approach, used historically’. Negative (No) responses reflect the species choice was ‘for molecule-specific reasons’. WT mouse is a wild-type strain of mouse; TG mouse is a transgenic mouse model.

**Figure 3:**
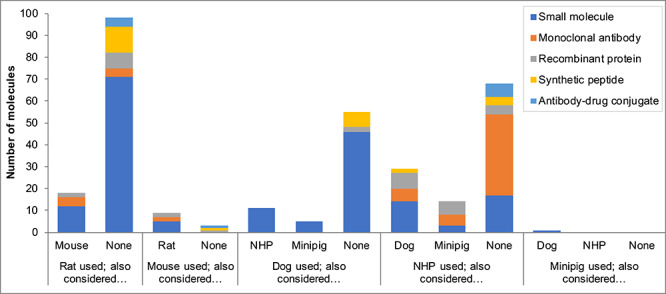
Which other species were considered during species selection? Note: The *x*-axis shows the species used within toxicology studies and answers to the follow-up question ‘Which other species were considered during species selection?’, where ‘considered’ was defined as ‘actively considered, i.e. tested for relevance’. ‘None’ reflects that no additional rodent species were considered (when a rodent was used), or separately that no additional non-rodent species were considered (when a non-rodent was used). Multiple additional species could be selected.

When the rat and dog were selected for small molecule testing, these tended to be the standard company practice (defined as the normal approach, used historically; Fig. 2) and often, no other species were considered (Fig. 3). When mouse or NHP were selected, these tended to be for case-by-case molecule-specific reasons and other species were also considered during these decisions (Fig. 3). For mAb testing, most (75%) that selected the NHP stated this was the standard company practice (as a pharmacologically relevant species) and for 84% of the mAbs, no other non-rodent species were considered. For testing synthetic proteins, the selection of rat and either dog or NHP were stated as standard company practice and generally no other species were considered during these decisions. For recombinant proteins, most (77%) selecting the NHP stated this was for molecule-specific reasons and other non-rodent species were considered during these decisions. Although the minipig was used for only one molecule within the dataset (a small molecule), this species was considered as a potential non-rodent for toxicology testing for some of the small molecules, mAbs and recombinant proteins*.*

**Figure 4:**
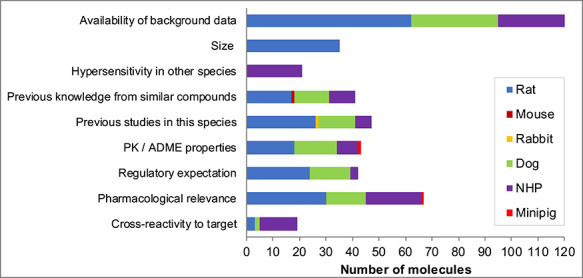
Which factors contributed to the species choice? Note: For each molecule, the factors contributing to the species choice were provided. Multiple factors could be selected. Size relates to the use of rat instead of mouse (for blood volume purposes). Regulatory expectation was defined as ‘an expectation that this species was required (perhaps from previous experience with similar compounds, or literature information), but in the absence of formal discussion or request from regulatory authorities’.

The factors contributing to selection of the rat and dog (all drug modalities combined) were primarily the availability of background data, previous studies in the species or knowledge from similar compounds and regulatory expectation (Fig. 4). Additional factors selected for mAbs, synthetic peptides, recombinant proteins and ADCs were pharmacological relevance and PK/ADME; these reasons were also selected for small molecules where species other than rat or dog were considered. The factors taken into account when NHP was selected were primarily cross-reactivity to target, pharmacological relevance, PK/ADME properties, hypersensitivity in other species and availability of background data, reflecting pertinent tests to justify the species for either biotherapeutic or small molecule testing.

Overall, the data show that regardless of the drug modality, there are multiple factors that contribute to species selection for toxicology studies and demonstration of species relevance. Even when the selected species is the ‘standard’ species used within the company for the specific drug modality, this does not mean that this selection is decided without adequate assessment of suitability. It is important to note that the phrase ‘standard company practice’ is likely to mean different things to different companies (and survey responders). For example, some companies may evaluate a specific ‘standard’ species first (by default), such as the rat and dog for small molecules or NHP for mAbs—particularly if these fall within a well-established class of molecules for the company; if the species is considered appropriate, then there may not be a need to evaluate other species. Other companies may evaluate a number of species/strains for comparison, which is considered their standard practice*.* These differences in working practices (and/or interpretation of the definition provided for ‘standard company practice’) between the participating companies have likely contributed to the variation in responses regarding consideration of other species during decision-making. Where NHP was the selected toxicology species for small molecule testing, there was evidence that this was not a standard practice (i.e. there were molecule-specific reasons for choice of NHP) and that other non-rodent species had also been considered. This was also the case for a proportion of NHPs used for mAb and other biotherapeutics testing. Even when the NHP was stated as the standard species for these drug modalities, multiple factors contributed to these decisions. This reflects the additional scientific justification that is required by many regional ethical review committees, before permitting the use of NHPs. For a subset of 17 small molecules using NHP, it was stated that no other non-rodent species had been considered during the decision-making. However, the factors described for selection of NHP did include hypersensitivity in other species or known effects from other studies/previous compounds that may preclude the usefulness of other non-rodents, thereby providing sufficient scientific justification for the use ofNHP.

The species used within the NC3Rs/ABPI dataset (all unmarketed products) were compared to publicly available data for products recently approved by the European Medicines Agency (EMA). Data published within European Public Assessment Reports (EPARs) were accessed on-line [30] for all 55 small molecules and 23 mAbs authorized by the EMA between 2016 and 2019 and mined to collate information for the species used in short-term (2–13 week) and long-term (26–52 week) general toxicology studies. For small molecules (Table 1), short-term toxicology studies were generally conducted in both rodent and non-rodent species, commonly rat and dog (16 small molecules) or rat and NHP (12 small molecules). However, a similar proportion (24 small molecules) used three or more species for short-term toxicity studies, typically with the addition of the mouse (21 small molecules) whilst 6 small molecules used both dog and NHP. The reasons for conducting toxicity testing in more than two species were not included within the EPARs and were a clear difference to the 92 small molecules reported in the NC3Rs/ABPI dataset [17]. Interestingly, 22 of the EPAR small molecules that used three or more species progressed to long-term studies, with 20 reducing to rat and either dog or NHP, another using rabbit and NHP and another retaining use of three species. Although not stated in the EPARs, the short-term mouse toxicity studies may be range-finders for carcinogenicity studies, with studies up to 13 week duration considered sufficient. The EPAR dataset provided examples of two small molecules (for neonatal diabetes mellitus or hypotension) where published *in vivo* data were used instead of conducting new studies, supporting extensions in additional populations (Table 1). It was common for mAb toxicity studies to be performed using NHP; 12 mAbs used the cynomolgus monkey only, a further 5 used a rodent species in addition and another 3 used a second non-rodent species (rabbit, rhesus macaque or chimpanzee) in addition for short-term toxicity testing (Table 2). Although not stated in the EPAR, it is likely the chimpanzee study was conducted prior to changes in acceptance for studies using this great ape species. Mouse (wild-type) was the only species used to test 2 mAbs, whilst *in vivo* toxicity studies were not performed for another mAb as no relevant animal species or valid transgenic mouse models were identified, with *in vitro* toxicity data provided instead (Table 2).

**Table 1:** The toxicology species used within small molecule packages approved by EMA (2016–2019)

Active substance (brand name)	Indication	Year	Short-term studies^a^	Long-term studies^b^
Alectinib (Alecensa)	ALK-positive NSCLS	2016	Rat; NHP	N/A
Avibactam sodium (Zavicefta)	Complicated infections	2016	Rat; Dog	N/A
Baricitinib (Olumiant)	Rheumatoid arthritis	2016	Mouse; Rat; Dog	Rat; Dog
Elbasvir/grazoprevir (Zepatier)	HCV genotypes 1 and 4	2016	Mouse; Rat; Dog	Rat; Dog
Eluxadoline (Truberzi)	Irritable bowel syndrome	2016	Mouse; Rat; NHP	Rat; NHP
Etelcalcetide (Parsabiv)	Secondary hyperparathyroidism	2016	Mouse; Rat; Dog	Rat; Dog
Ixazomib citrate (Ninlaro)	Multiple myeloma	2016	Rat; Dog	Rat; Dog
Migalastat hydrochloride (Galafold)	Fabry disease	2016	Mouse; Rat; Dog; NHP	Rat; NHP
Obeticholic acid (Ocaliva)	Primary biliary cholangitis	2016	Mouse; Rat; Dog	Rat; Dog
Opicapone (Ongentys)	Parkinson’s disease	2016	Mouse; Rat; Dog; NHP	Rat; NHP
Palbociclib (Ibrance)	Breast cancer	2016	Rat; Dog	Rat; Dog
Selexipag (Uptravi)	Pulmonary hypertension	2016	Mouse; Rat; Dog	Rat; Dog
Sofosbuvir/velpatasvir (Epclusa)	Chronic HCV infection	2016	Mouse; Rat; Dog	Rat; Dog
Trifluridine/tipiracil (Lonsurf)	Metastatic colorectal cancer	2016	Rat; NHP	N/A
Venetoclax (Venclyxto)	Chronic lymphocytic leukaemia	2016	Mouse; Dog	Mouse; Dog
Cariprazine (Reagila)	Schizophrenia	2017	Rat; Dog	Rat; Dog
Fluciclovine (Axumin)	Prostate cancer	2017	Rabbit; Rat; Dog	N/A
Letermovir (Prevymis)	Prophylaxis of cytomegalovirus reactivation	2017	Mouse; Rat; NHP	Rat; NHP
Midostaurin (Rydapt)	Acute myeloid leukaemia	2017	Mouse; Rat; Dog; NHP	Rat; Dog
Niraparib (Zejula)	Ovarian, fallopian tube, or peritoneal cancers	2017	Rat; Dog	N/A
Padeliporfin dipotassium (Tookad)	Prostate cancer	2017	Rat; NHP	N/A
Patiromer sorbitex calcium (Veltassa)	Hyperkalaemia	2017	Rat; Dog	Rat; Dog
Ribociclib (Kisqali)	Breast cancer	2017	Rat; Dog	Rat; Dog
Semaglutide (Ozempic)	Type 2 diabetes mellitus	2017	Rat; NHP	Rat; NHP
Sodium zirconium cyclosilicate (Lokelma)	Hyperkalaemia	2017	Rat; Dog	Rat; Dog
Telotristat (Xermelo)	Metastatic neuroendocrine tumours	2017	Mouse; Rat; Dog	Rat; Dog
Tivozanib HCl monohydrate (Fotivda)	Renal cell carcinoma	2017	Rat; NHP	N/A
Tofacitinib (Xeljanz)	Rheumatoid arthritis	2017	Rat; NHP	Rat; NHP
Abemaciclib (Verzenios)	Breast cancer	2018	Rat; Dog	N/A
Binimetinib (Mektovi)	Melanoma	2018	Rat; NHP	Rat; NHP
Brexpiprazole (Rxulti)	Schizophrenia	2018	Mouse; Rat; NHP	Rat; NHP
Brigatinib (Alunbrig)	NSCLC	2018	Rat; NHP	Rat; NHP
Doravirine (Delstrigo)	HIV 1	2018	Mouse; Rat; Dog	Rat; Dog
Doravirine (Pifeltro)	HIV 1	2018	Mouse; Rat; Dog	Rat; Dog
Encorafenib (Braftovi)	Melanoma	2018	Rat; NHP	N/A
Eravacycline dihydrochloride (Xerava)	Complicated intra-abdominal infections	2018	Rat; Dog; NHP	Rat; Dog; NHP
Ertugliflozin (Segluromet)	Type 2 diabetes mellitus	2018	Mouse; Rat; Dog	Rat; Dog
Ertugliflozin (Steglatro)	Type 2 diabetes mellitus	2018	Mouse; Rat; Dog	Rat; Dog
Ertugliflozin (Steglujan)	Type 2 diabetes mellitus	2018	Mouse; Rat; Dog	Rat; Dog
Glibenclamide (Amglidia)	Neonatal diabetes mellitus	2018	No new studies	N/A
Ivacaftor (Symkevi)	Cystic fibrosis	2018	Mouse; Rat; Dog	Rat; Dog
Neratinib (Nerlynx)	Breast Cancer	2018	Rat; Dog	Rat; Dog
Peramivir (Alpivab)	Influenza	2018	Rat; NHP	Rat; NHP
Rucaparib (Rubraca)	Ovarian cancer	2018	Rat; Dog	Rat; Dog
Angiotensin II acetate (Giapreza)	Hypotension	2019	No new studies	N/A
Avatrombopag (Doptelet)	Severe thrombocytopenia	2019	Mouse; Rat; Dog; NHP	N/A
Dacomitinib (Vizimpro)	Metastatic NSCLC	2019	Rat, Dog	Rat, Dog
Delafloxacin (Quofenix)	Acute Bacterial Skin Infections	2019	Rat; Dog	Rat; Dog
Gilteritinib (Xospata)	Acute myeloid leukaemia	2019	Rat; Dog	N/A
Larotrectinib (Vitrakvi)	Solid tumours with NTRK gene fusion	2019	Rat; NHP	N/A
Netarsudil (Rhokiinsa)	Elevated intraocular pressure	2019	Rabbit; Rat; Dog; NHP	Rabbit; NHP
Pegvaliase (Palynziq)	Phenylketonuria	2019	Rat; NHP	Rat; NHP
Siponimod (Mayzent)	Multiple sclerosis	2019	Mouse; Rat; NHP	Rat; NHP
Sotagliflozin (Zynquista)	Type 1 diabetes mellitus	2019	Rat; Dog	Rat; Dog
Upadacitinib (Rinvoq)	Rheumatoid arthritis	2019	Rat; Dog	Rat; Dog

N/A indicates these studies were not described within the EPAR. Additional species used for developmental and reproductive toxicity studies are not included in the table. HIV, human immunodeficiency virus; NSCLS, non-small cell lung cancer; NTRK, Neurotrophic Tyrosine Receptor Kinase. NHP is the cynomolgus monkey and mouse is a wild-type strain for all cases. Where mouse is included as a toxicology species, this was for general toxicity studies of at least 2-week duration; although not stated within the EPARs these may have been performed as dose-range finding studies for a carcinogenicity study. Where mouse was used for acute (single-dose) toxicity studies this was not included in the table.

^a^The toxicology species used for pivotal GLP toxicology studies to supportFIH.

^b^Phase II/III

**Table 2:** The toxicology species used within mAb packages approved by EMA (2016–2019)

Active substance (brand name)	Indication	Year	Short-term studies^a^	Long-term studies^b^
Bezlotoxumab (Zinplava)	*C. difficile* infection	2016	Mouse	N/A
Daratumumab (Darzalex)	Multiple myeloma	2016	Chimpanzee; NHP	N/A
Elotuzumab (Empliciti)	Multiple myeloma	2016	None	None
Ixekizumab (Taltz)	Plaque psoriasis	2016	NHP	NHP
Olaratumab (Lartruvo)	Soft tissue sarcoma	2016	NHP	NHP
Reslizumab (Cinqaero)	Asthma	2016	Mouse; NHP	Mouse; NHP
Atezolizumab (Tecentriq)	Metastatic urothelial carcinoma	2017	Mouse; NHP	NHP
Avelumab (Bavencio)	Metastatic Merkel cell carcinoma	2017	Mouse; Rat; NHP	NHP
Benralizumab (Fasenra)	Severe asthma	2017	NHP	NHP
Brodalumab (Kyntheum)	Plaque psoriasis	2017	NHP	NHP
Burosumab (Crysvita)	X-linked hypophosphataemia	2017	Rabbit; NHP	NHP
Dupilumab (Dupixent)	Atopic dermatitis	2017	NHP	NHP
Ocrelizumab (Ocrevus)	Multiple sclerosis	2017	NHP	NHP
Sarilumab (Kevzara)	Rheumatoid arthritis	2017	NHP	NHP
Durvalumab (Imfinzi)	NSCLC	2018	NHP	N/A
Erenumab (Aimovig)	Migraine	2018	NHP	N/A
Emicizumab (Hemlibra)	Haemophilia A	2018	NHP	NHP
Lanadelumab (Takhzyro)	Hereditary angioedema	2018	Rat; NHP	NHP
Tildrakizumab (Ilumetri)	Psoriasis	2018	NHP	NHP
Fremanezumab (Ajovy)	Migraine	2019	Rat; NHP	NHP
Ibalizumab (Trogarzo)	Multidrug resistant HIV-1	2019	NHP^RH^; NHP	NHP
Ravulizumab (Ultomiris)	PNH	2019	Mouse	Mouse
Risankizumab (Skyrizi)	Plaque psoriasis	2019	NHP	NHP

N/A indicates these studies were not described within the EPAR. A single toxicology study of 3 months duration is acceptable for immune-oncology pharmaceuticals, as described in ICHS9 [10]. NSCLS, non-small cell lung cancer; PNH, paroxysmal nocturnal haemoglobinuria. NHP is the cynomolgus monkey; NHP^RH^ is rhesus macaque; Mouse is a wild-type strain for all cases. Where mouse is included as a toxicology species, this was for general toxicity studies of at least 2-week duration. Additional species used for developmental and reproductive toxicity studies are not included in the table.

^a^The toxicology species used for pivotal GLP toxicology studies to supportFIH.

^b^Phase II/III

In the NC3Rs/ABPI dataset, 2 out of 6 mAbs that used two species for short-term studies adopted the accepted ICHS6 (R1) approach to reduce to a single species, with scientific justification, for longer term studies; in both cases, the rodent was progressed [17]. Within the EPAR dataset, 6 out of 7 mAbs where two species were used for short-term studies reduced to one species for the longer term studies; however, in all cases the rodent was not progressed, leading to continued use of NHPs. The NC3Rs/ABPI dataset also included one example of a small molecule that adopted a similar approach, using dog only for longer term studies [17].

Our review of publicly available regulatory documents for information on species use and justification identified limitations due to wide variability in the level of detail to enable this type of data to be collated; for some submissions it proved impossible to determine the durations of toxicity studies performed and many did not include justification for species selection (as previously noted by others [31]). Future, routine inclusion of such information for all drug modalities would help to improve transparency and understanding of species decisions for reviewers and, once published, for external parties. Our analysis of publicly available regulatory documents is also inherently biased as only those molecules that successfully complete development with a successful marketing application are included. Nevertheless, comparison of the information gleaned from the recent EPAR dataset with the NC3Rs/ABPI dataset does show that there are common approaches for the selection of species across the industry, as expected from similar experiences of drug development and the limited range of toxicology species commonly used. Furthermore, variation in factors leading to decisions and the presence of ‘atypical’ or molecule-specific approaches in both datasets indicate that species selection is not a ‘tick-box’ decision and that the use of a ‘standard’ species is not an unconsidered decision. It should also be acknowledged that target organ findings in the toxicity studies themselves often provide some vindication that the rodent and/or non-rodent species were suitable and/or relevant.

## Are non-human primates always the species of choice for development of biologics? (*Alison Wolfreys, UCB)*

The generic term ‘biologics’ refers to the large number and varied classes of ‘large molecule’ drugs, including mAbs, vaccines, synthetic peptides and nucleotides, recombinant proteins or peptides, gene and cell therapies and other blood and tissue products. Even the classical first-generation mAb scaffolds have evolved into other specific formats such as bispecifics, Fabs, ADCs etc. The main regulatory guideline that applies to these products is ICHS6 (R1), used in conjunction with ICHM3 (R2). The key difference between these guidelines is the wording in relation to species selection for toxicity testing, where toxicity studies should only be conducted with ‘pharmacologically relevant’ species described within ICHS6 (R1), in contrast to the general ‘rodent and non-rodent’ species recommended within ICHM3(R2). With toxicity studies in non-relevant species actively discouraged, the onus is on the developing company to provide data demonstrating high potency and specificity for the target in the test species, as well as comparable downstream effects of target inhibition, which often prevents use of the more common rodent and non-rodent species such as rat and dog*.* In the past, when many novel biologics targeted molecules such as those in the species-diverse immunology pathways, the NHP was frequently the only pharmacologically relevant species and this sometimes led to the assumption that the NHP is the only relevant species to be used by default. However, if targeting evolutionarily conserved targets and pathways (such as those in fibroblasts, osteoblasts, etc.), there are often other relevant species and a rodent plus non-rodent are required for the toxicology programme to support First in human (FIH) clinical trials. The NC3Rs/ABPI project discussed above showed that two species were used for toxicology testing of 30% mAbs and the majority of other biologics [17], whilst a review of 39 mAbs submitted in Japan up to 2016 indicated that short-term toxicity studies were conducted in two species for 41% of mAbs [16]. To ensure the most relevant species are used for toxicity testing, potential species should be assessed for pharmacological relevance in addition to NHP and at UCB, the cynomolgus monkey, rat and mouse are usually screened for each biologic. Dog and minipig are screened on a more case-by-case basis, particularly as many biologics are highly immunogenic in the dog and this often precludes their use in repeat dose toxicology studies.

There are a number of ways in which a species can be shown to be pharmacologically relevant, with the most common described below.

(1) High target sequence homology to human: the gene sequence of the target is compared between non-clinical species and human [32]. The full genomic sequence of all the common toxicology species is available [33] and in general, the lower the percentage homology, the less likely to translate into pharmacological activity. This may be sufficient to discount some species.
(2) Similar target binding affinity to the human and toxicology species target: The difference in potency between the toxicology species and the human should ideally be less than 10-fold. A number of *in vitro* assessments can be performed, whereby BIAcore is typically conducted first, to determine the binding affinity (Kd) of the biologic to the target protein, as this can often rapidly remove several species under consideration. The more physiological assays are then conducted, based on need and assay availability. These include ELISA or fluorescence-activated cell sorting to determine binding of the target in a cellular matrix and/or cell-based assays to determine the relative IC_50_/IC_90_ or EC_50_/EC_90_ values using a pharmacodynamic (PD) endpoint.
(3) Similar target expression and distribution to human, by comparison of cell types and tissues for expression of the target, using Microarray and RNAseq data from databases such as Genecards or immunohistochemistry of target binding profiles. If the target is only expressed in diseased states or the target is not expressed in a candidate test species, then the test species is unlikely to be a useful model as there is potential for missed toxicities. Conversely, if the target is expressed in cell types and/or organs in one or more of the candidate test species but not in the human, there is potential to generate irrelevant toxicities.
(4) Achievable target occupancy and target kinetics, to compare the expected potency in human *versus* candidate test species using modelling and simulation techniques. If the biologic is significantly less potent in the candidate species than in human, it may not be possible to dose enough to achieve high levels of target occupancy for an efficacious effect. Turnover of the target is also important, as a short half-life would require higher potency, higher dose and more frequent dosing to offset rapid restoration of the target.
(5) Functional equivalence of PD effects *in vivo*, to provide sufficient knowledge of the mode of action (MoA) to understand if the candidate species will predict (over or under prediction) potential human effects. Rituximab (a mAb against CD20 expressed on B cells) exemplifies a good correlation of PD effects between species, with the expected decrease in B cell counts demonstrated in mice with a similar time-course as humans [34]. TGN1412 (a CD28 super-agonist mAb) provides a poor correlation of PD effects between species, with a lack of understanding of the different expression and downstream signalling between humans and NHPs, which ultimately led to extensive, life-threatening toxicities in the first patients dosed [35] due to a severe Cytokine Release Syndrome (Cytokine storm). However, once understood, the molecule re-entered clinical development [36]. Determination of cytokine release is now standard for high-risk biologics [37].

On occasion, there are no pharmacologically relevant test species identified, as the biologic does not bind with the target in any species. In such cases, the use of transgenic models expressing human receptors or homologous proteins can be considered [14]. Alternatively, there may be no pharmacologically relevant test species when the biologic is directed at foreign targets such as bacterial or viral targets. For these specific cases, ICHS6 (R1) specifies that a short-term safety study in one species (as justified by the sponsor) can be considered (often rodent) and no additional toxicity studies, including reproductive toxicity studies, are appropriate.

If there are two pharmacologically relevant species identified (one rodent and one non-rodent), then both species should be used for short-term general toxicology studies supporting Phase I clinical trials. After this, toxicology testing can be conducted in the most sensitive species only, provided that toxicological findings are similar or the relative sensitivity of the two species is understood from the MOA. In practice, if rodents and NHPs have similar toxicities at the same or different doses, the species with findings at the lowest dose or with the most severe toxicities is likely to be selected for further testing (i.e. the most sensitive species). However, the rodent species should be considered unless there is a scientific rationale for using non-rodents, which might include the higher potential for rodents to be impacted by generation of anti-drug antibodies (ADA) as the humanized mAb is more likely to be identified as ‘foreign’ in rodents. The formation of ADA often leads to marked decrease/absence of drug in blood (enhanced clearance) which may invalidate studies. If the potential for ADA incidence is high, then rodent group sizes may need to be increased to allow sufficient animals to remain exposed to the biologic by the end of the study [38]. An example of a mAb antagonist of TGFβ1 where different toxicities were observed between rat and NHP is described elsewhere (Bio-3 [39]), whereby multiple toxicities were identified in NHP but none in rat, which would have likely led to selection of the NHP for longer term toxicity studies. An example of a mAb targeting FGF-23 (Burosumab) conducted short-term studies with rabbit and NHP, as rodents were not sensitive. Almost identical toxicities were observed, but only the NHP progressed into longer duration toxicity studies (Table 2).

In summary, biologics differ from small molecule entities as toxicology studies should only be conducted in a species which is pharmacologically relevant and testing in irrelevant species is actively discouraged. Although the NHP may often be the only pharmacologically relevant species for assessing the toxicology of a novel biologic, this is a proactive selection and not a default position. Demonstrating a species is pharmacologically relevant is more than just exhibiting that it is ‘active’, typically achieved by establishing at least two of the following: high target sequence homology to human, similar target binding affinity to human, similar target expression and distribution to human, achievable target occupancy and target kinetics and/or functional equivalence of PD effects. In some cases, particularly for highly conserved pathways or recombinant proteins, this will equate to two species toxicology testing. If two species are used in the initial toxicology testing, it is possible to use only the most toxicologically sensitive species in post-FIH-enabling studies if toxicity is similar/identical in both species or one species shows alternative reasons why it would not be an ideal choice, such as high incidence ofADA.

## When is the minipig the relevant non-rodent toxicology species? (*Richard Haworth, GSK)*

The use of minipig as the non-rodent species for regulatory testing has long been advocated [40] and there are aspects of their anatomy, physiology and biochemistry, which make this species suitable for consideration as a toxicology species. However, the use of the minipig remains low [41], potentially due to lack of inclusion at early screening stages and historical preference for other non-rodent species [21], with selection as the non-rodent species predominantly for short-term toxicology studies with dermal, oral and parenteral routes of administration [42]. Nevertheless, many companies are considering the minipig as an alternative to dogs for small molecule development [21, 22] and also to reduce the use of NHPs. Their use in biopharmaceutical development has been limited by a lack of background data, lack of PD relevance, lack of reagents or biomarkers, concerns regarding immune system characterization, potential for induction of ADAs and poor suitability for developmental toxicity assessments as a consequence of absence of placental antibody transfer in the minipig [40, 42]. There is increasing knowledge regarding the binding affinity of minipig Fc gamma receptors for human immunoglobulins and this information needs to be considered when selecting a non-rodent species which will most appropriately test a particular mechanism of human relevant toxicity associated with a particular therapeutic antibody [43].

GSK uses appropriate animal species for experimental studies in support of developing potential new medicines for clinical use, which is enabled by an objective assessment of the human-relevance of possible animal models. The relative merits of the dog and minipig as the most suitable non-rodent toxicology species are considered for every small molecule at an early point in development. For toxicology studies, selection of the non-rodent species is scientifically justified and documented, based on a weight of evidence approach; there is no default species selection, each molecule is assessed on a case-by-case basis. Human relevance requires sufficient concordance across a range of biological and pharmacologic attributes and NHPs are only used if the dog and the minipig have been ‘deselected’ (proved to be unsuitable). A science based, data-driven approach is applied using factors including pharmacological similarity (1 and 2 below), similarity of kinetics, disposition and drug metabolism (3 and 4 below) and compound and study specific criteria (5, 6, 7, below).

(1) Target homology and tissue-specific distribution compared with human. *In silico, in vitro*, transcriptomic and proteomic analysis and quantitative immunostaining are valuable in ascertaining the similarity of the target and its distribution in a given species relative to humans.
(2) PD responsiveness. The use of literature, *in vitro* and *in silico* data to determine the current state of knowledge of the degree of PD homology with humans, including that downstream signalling from the target/receptor is appropriate. Specific PD biomarkers may be useful to compare across species, using quantitative dose–response relationships. For biologics, expression of the receptor or an epitope that results in pharmacological activity must be demonstrated. Appropriate tests are utilized to assess target affinity and function to aid identification of relevant species. In addition, information on receptor/epitope distribution may provide some understanding of potential *in vivo* toxicity.
(3) Disposition and drug exposure. This is modelled using, for example, *in silico, in vitro* data, and if required, a comparative, single dose TK/PK study, frequently in the minipig and dog, and when scientifically necessary, in the NHP in addition. The species selected should have demonstrated likelihood to attain sufficient drug exposure to meet study objectives, e.g. supra-therapeutic exposure for toxicology studies.
(4) Metabolite profile. An *in vitro* metabolite cross species comparison, which can be complemented with *in vivo* animal data as required, to assess metabolite profiles (e.g. compare pattern of major metabolites to human using appropriate *in vitro* systems) to determine the species with the greatest human relevance.
(5) Physiological and toxicological human relevance. For example, where a species is known (via literature and/or experience) to have inappropriate or over sensitivity to a drug modality, molecular class or target, e.g. compounds likely to cause acute histamine release in dogs [44, 45].
(6) Study specific and animal welfare considerations. Species differ in their practical constraints and impact on study specific scientific objectives, i.e. technical feasibility, tolerability or comparative stress associated with special techniques—such as collection of lymph or cerebrospinal fluid.
(7) Historic background data. This is often used to provide context to interpret sample variation. Note, this will not be the determining factor in species selection.

All animal studies in the following case studies were ethically reviewed and carried out in accordance with Animals (Scientific Procedures) Act 1986 and the GSK Policy on the Care, Welfare and Treatment of Animals.

### Case study 1

A small molecule, innate immune system agonist, evaluated in dog, minipig and NHP. The pharmacological responses are induction of Interferon (IFN)-α (the clinical marker of efficacy) and Tumour Necrosis Factor (TNF)-α (the clinical marker of safety)—these were evaluated in minipig and NHP using *in vitro* assays, with the minipig assays developed in-house as they were unavailable commercially. As very low doses were expected to be required, systemic drug and metabolite profiles were not considered likely to be decisive in species selection. In early oral or intravenous PK studies evaluating three other small molecules directed at the same target, the dog was shown to poorly tolerate the test molecule (producing lethargy, subdued behaviour and emesis) and was discounted from future studies. Comparison of TNF-α and IFN-α responses in human and cynomolgus monkey whole blood or peripheral blood mononuclear cells (PBMC) cultured *in vitro* with the test article showed that the relative potencies in the two species were very similar (unpublished data). The minipig showed comparable TNF-α results with human; however, levels of IFN-α were significantly lower. There was also poor selectivity for IFN-α compared to TNF-α indicating that the mini-pig was not suitable for use as the non-rodent test species. In contrast, the cynomolgus monkey had been shown to produce comparable cytokine profiles for both IFN-α and TNF-α, with similar selectivity for IFN-α over TNF-α. As the cynomolgus monkey exhibited a more similar pharmacological response to humans than that seen for minipigs, the cynomolgus monkey was selected as the non-rodent toxicology species. This represents an example where the PD responsiveness was a key factor in the rational selection of the non-rodent species.

### Case study 2

A small molecule for an undisclosed indication, given by the oral dose route against a drug target which is well conserved across the non-rodent toxicology species. This compound was initially evaluated in the dog to enable comparability with a previous compound in the same pharmacological class which had used dog as the test species. However, emesis was a potential risk identified from knowledge of the compound class and pharmacology. To ensure the species choice decision was based on evidence, a 3-day tolerability study in the dog was conducted in which emesis was confirmed. This led to non-linear TK at higher doses and the dog was deemed unsuitable as the toxicology species. The same dose given to minipigs was tolerated, providing sufficient drug exposure relative to anticipated clinical dose for continuation as the non-rodent toxicology species. This represents an example where the tolerability and secondary effects on toxicokinetics was decisive in the species selected.

### Case study 3

A small molecule was in development for a subcutaneous clinical indication, requiring dosing in the toxicology programme by the same route. The minipig was selected as the non-rodent species and provided satisfactory PK; however, the clinical route changed to intravenous infusion and toxicology studies via that route (infusion via ear vein) encountered procedural dosing issues and poor tolerability in the minipigs. When the dog was used for subsequent studies, the intravenous infusion dosing was tolerated. This provides a good example of application of study specific and animal welfare considerations to ensure the appropriate species is chosen to enable successful dosing.

In certain cases, it will not be necessary to consider data on all of the factors; for instance, if there is an overwhelming difference between the toxicological or physiological relevance of dog and minipig. However, to determine if the minipig is the most relevant non-rodent toxicology species for a particular project, requires the responsible decision maker to carefully evaluate the factors influencing species selection (listed above) and determine on a weight of evidence approach.

## Concluding remarks

The pharmaceutical industry is actively working to provide alternative methodologies to decrease reliance on animal studies and to improve predictivity and safety assessments. Whilst toxicity studies in animals remain a current regulatory requirement, there is a responsibility to ensure the 3Rs are applied and that any data generated is relevant and used to minimize risk of adverse events in humans. However, it is acknowledged that a high proportion of potential new medicines fail to reach marketing authorization and that many thousands of animals may have been used for toxicity testing leading up to those decisions. The reasons for pharmaceutical attrition and predictivity of animal toxicity tests are not the focus of this publication, nevertheless, appropriate species selection could influence attrition in a positive direction by enabling early progression or termination decisions based on confidence in human relevance. The result would be a higher chance of success of drug compounds as they progress through development with reduced late stage attrition. Additional areas where species selection is directly applicable to the 3Rs are outlined below:

(1) Use of *in vitro* and *in silico* approaches early within drug discovery to investigate liabilities of concern and reduce potential for adverse findings for drug candidates that do progress to animal studies.
(2) Inclusion of a wider range of toxicology species in selection criteria, where there are no scientifically justifiable reasons for selecting a particular species, e.g. selecting a preferred species used historically for a specific drug modality. Importantly, evidence that several potential species were assessed for relevance early within the project, and inclusion of the data within regulatory submissions, forms an important part of the justification for species choice that may reduce (or avoid) questions and potential requests to perform studies in additional species later in development.
(3) Demonstrating that there is no pharmacologically relevant species for biologics can lead to an overall reduction in animal use if *in vitro*-only packages prove to be acceptable for marketing authorization. There is evidence that these approaches are being used where necessary in the immune-oncology field, e.g. when a transgenic model is not available/possible (Table 2) [46, 47].
(4) Identification of a pharmacologically relevant rodent species for biologics allows early pharmacology work to be conducted in this species with the clinical candidate. Both a rodent and non-rodent would be required for short-term toxicity testing to support FIH clinical trials but, if similar toxicities were apparent in both species, the rodent only may be progressed to longer term studies, reducing the use of NHPs. As previously reported, this approach would also reduce animal use if applied more widely to other drug modalities [17].
(5) Use of pharmacologically irrelevant species for testing of novel biologics is actively discouraged as this could lead to the generation of irrelevant toxicology data, which could stop development of a potentially useful new drug. Conversely, the generation of false negative data may fail to detect toxicities that are later apparent in humans. In both cases, a lack of human-relevant data at an appropriate stage of development can undermine sound project decisions. This can compromise the intended patient benefit of the development project and may lead to further animal use in an attempt to understand any toxicities observed and their relevance to the human.

Justification for species choice is the responsibility of the company developing the new pharmaceutical, with the number of species assessed, the specific tests performed and weighting of other factors likely to be a company-specific process, dependent upon different experiences within the industry and of different drug modalities, target class and/or chemical class involved. Little information is available for how these decisions are made, with minimal details included within regulatory submissions for molecules that progress that far. At this point, the studies are complete and animals have been used, therefore, it is unlikely that regulators will request studies in different species unless there is a critical flaw in the package of toxicology studies that would preclude use in the clinic. The data included herein from the NC3Rs/ABPI project and examples of current practice and processes from two pharmaceuticals companies are a rare insight into these decisions, in the absence of other literature on this topic.

It is clear from the presentations summarized above that selection of the second species for toxicology testing, and in some circumstances, selection of any species for toxicology testing, should be a carefully considered, proactive decision and not a default to a preferred species. Multiple factors drive this decision, including demonstration of pharmacological relevance, species relevance for the expected pharmacology, modality, chemical/target class, PK profile, PD responses, animal welfare considerations and incorporation of the 3Rs into the overall toxicology package. In addition, there are likely differences between companies on how these factors are evaluated and the weighting given to each one. However, the over-riding principle is that selection of the species should be underpinned by a thoroughly evaluated scientific rationale. For biologics, toxicology testing is restricted to pharmacologically relevant species only, which may result in use of a single species only. Even if two species are used in the initial toxicology studies, it is possible to reduce to one species for the longer term toxicology studies if the toxicological profile is identical/similar in both species. This is not yet an option provided in the regulatory guidelines for small molecules, but this may become a possibility in the future.

## References

[1] https://www.ich.org/page/safety-guidelines (1 July 2020, date last accessed).

[2] Goh J-Y, Weaver R, Dixon L et al. Development and use of in vitro alternatives to animal testing by the pharmaceutical industry 1980–2013. Toxicol Res 2015;4:1297–1307.

[3] Kendrick J, Stow R, Ibbotson N et al. A novel welfare and scientific approach to conducting dog metabolism studies allowing dogs to be pair-housed. Lab Anim 2020, doi: 10.1177/0023677220905330, online ahead of print. 32063096

[4] Prior H, Bottomley A, Champeroux P et al. Social-housing of non-rodents during telemetry recordings in safety pharmacology and toxicology studies. J Pharmacol Toxicol Methods 2016;81:75–87. 27039257 10.1016/j.vascn.2016.03.004 PMC5056765

[5] Spooner N, Anderson K, Siple J et al. Microsampling: considerations for its use in pharmaceutical drug discovery and development. Bioanalysis 2019;11:1015–1038. 31218897 10.4155/bio-2019-0041

[6] Chapman K, Holzgrefe H, Black L et al. Pharmaceutical toxicology: designing studies to reduce animal use, while maximizing human translation. Regul Toxicol Pharmacol 2013;66:88–103. 23524271 10.1016/j.yrtph.2013.03.001

[7] Redfern WS, Ewart L, Lainée P et al. Functional assessments in repeat-dose toxicity studies: the art of the possible. Toxicol Res 2013;2:209–234.

[8] Sewell F, Chapman K, Baldrick P et al. Recommendations from a global cross-company data sharing initiative on the incorporation of recovery phase animals in safety assessment studies to support first-in-human clinical trials. Regul Toxicol Pharmacol 2014;70:413–429. 25078890 10.1016/j.yrtph.2014.07.018

[9] ICHM3 (R2) Nonclinical safety studies for the conduct of human clinical trials and marketing authorization for pharmaceuticals In: International Conference on Harmonisation (ICH). Topic M3(R2). 2009. 20349552

[10] ICHS9 Nonclinical evaluation for anticancer pharmaceuticals In: International Conference on Harmonisation (ICH). Topic S9. 2010.

[11] Baldrick P Safety evaluation to support first-in-man investigations II: toxicology studies. Regul Toxicol Pharmacol 2008;51:237–243. 18501490 10.1016/j.yrtph.2008.04.006

[12] Butler L, Guzzie-Peck P, Hartke J et al. Current nonclinical testing paradigms in support of safe clinical trials: an IQ consortium DruSafe perspective. Regul Toxicol Pharmacol 2017;87:S1–S15. 10.1016/j.yrtph.2017.05.009 28483710

[13] Monticello TM, Jones T, Dambach D et al. 2017 Current nonclinical testing paradigm enables safe entry to first-in-human clinical trials: the IQ consortium nonclinical to clinical translational database. Toxicol Appl Pharmacol 334:100–109. 28893587 10.1016/j.taap.2017.09.006

[14] ICHS6(R1), 2011 Preclinical safety evaluation of biotechnology-derived pharmaceuticals In: International Conference on Harmonisation (ICH). Topic S6(R1). 22616137

[15] Brennan FR, Cauvin A, Tibbitts J et al. Optimized nonclinical safety assessment strategies supporting clinical development of therapeutic monoclonal antibodies targeting inflammatory diseases. Drug Dev Res 2014;75:115–161. 24782266 10.1002/ddr.21173

[16] Iwasaki K, Uno Y, Utoh M et al. Importance of cynomolgus monkeys in development of monoclonal antibody drugs. Drug Metab Pharmacokinet 2019;34:55–63. 29655914 10.1016/j.dmpk.2018.02.003

[17] Prior H, Baldrick P, Beken S et al. Opportunities for use of one species for longer-term toxicology testing during drug development: a cross-industry evaluation. Regul Toxicol Pharmacol 2020;113:104624. 32126256 10.1016/j.yrtph.2020.104624

[18] EMEA/CHMP/SWP/28367/07 Rev. 1 Guideline on Strategies to Identify and Mitigate Risks for First-in-Human and Early Clinical Trials with Investigational Medicinal Products. 2017. 10.1111/bcp.13550 PMC6005602 29451320

[19] Grimes J, Desai S, Charter N et al. MrgX2 is a promiscuous receptor for basic peptides causing mast cell pseudo-allergic and anaphylactoid reactions. Pharmacol Res Perspect 2019:7;547. 10.1002/prp2.547 PMC6887720 31832205

[20] Sert N du, Holmes A, Wallis R et al. Predicting the emetic liability of novel chemical entities: a comparative study. Br J Pharmacol 2012;165:1848–1867. 21913900 10.1111/j.1476-5381.2011.01669.x PMC3372835

[21] Jones K, Harding J, Makin A et al. Perspectives from the 12th annual minipig research forum: early inclusion of the minipig in safety assessment species selection should be the standard approach. Toxicol Pathol 2019;47:891–895. 31280706 10.1177/0192623319861940

[22] Schaefer K, Rensing S, Hillen H et al. Is science the only driver in species selection? An internal study to evaluate compound requirements in the minipig compared to the dog in preclinical studies. Toxicol Pathol 2016;44:474–479. 26839331 10.1177/0192623315624572

[23] 2010/63/EU Directive 2010/63/EU of the European Parliament and of the Council of 22 September 2010 on the Protection of Animals Used for purposes.

[24] Vermeire T, Epstein M, Badin RA et al. Final opinion on the need for non-human primates in biomedical research, production and testing of products and devices (update 2017) In: Scientific Committee on Health, Environmental and Emerging Risks (SCHEER)*.* Brussels: European Commission, 2017.

[25] Clark M, Steger-Hartmann T. A big data approach to the concordance of the toxicity of pharmaceuticals in animals and humans. Regul Toxicol Pharmacol 2018;96:94–105. 29730448 10.1016/j.yrtph.2018.04.018

[26] Olson H, Betton G, Robinson D et al. Concordance of the toxicity of pharmaceuticals in humans and in animals. Regul Toxicol Pharmacol 2000;32:56–67. 11029269 10.1006/rtph.2000.1399

[27] Bailey J, Balls M. Recent efforts to elucidate the scientific validity of animal-based drug tests by the pharmaceutical industry, pro-testing lobby groups, and animal welfare organisations. BMC Med Ethics 2019;20:16. 30823899 10.1186/s12910-019-0352-3 PMC6397470

[28] Meer PJ van, Kooijman M, Gispen-de Wied C et al. The ability of animal studies to detect serious post marketing adverse events is limited. Regul Toxicol Pharmacol 2012;64:345–349. 22982732 10.1016/j.yrtph.2012.09.002

[29] Prior H, Baldrick P, Hann L de et al. Reviewing the utility of two species in general toxicology related to drug development. Int J Toxicol 2018;37:121–124.

[30] https://www.ema.europa.eu (1 July 2020, date last accessed).

[31] Baldrick P Getting a molecule into the clinic: nonclinical testing and starting dose considerations. Regul Toxicol Pharmacol 2017;89:95–100. 28751261 10.1016/j.yrtph.2017.07.027

[32] BLAST: Basic Logic Alignment Search Tool https://blast.ncbi.nlm.nih.gov/Blast.cgi (1 July 2020, date last accessed).

[33] Vamathevan J, Hall M, Hasan S et al. Minipig and beagle animal model genomes aid species selection in pharmaceutical discovery and development. Toxicol Appl Pharmacol 2013;270:149–157. 23602889 10.1016/j.taap.2013.04.007

[34] Häusler D, Häusser-Kinzel S, Feldmann L et al. Functional characterization of reappearing B cells after anti-CD20 treatment of CNS autoimmune disease. Proc Natl Acad Sci 2018;115:9773–977. 30194232 10.1073/pnas.1810470115 PMC6166805

[35] Suntharalingam G, Perry M, Ward S et al. Cytokine storm in a phase 1 trial of the anti-CD28 monoclonal antibody TGN1412. N Engl J Med 2006;355:1018–1028. 16908486 10.1056/NEJMoa063842

[36] Tyrsin D, Chuvpilo S, Matskevich A et al. From TGN1412 to TAB08: the return of CD28 superagonist therapy to clinical development for the treatment of rheumatoid arthritis. Clin Exp Rheumatol 2016;34:S45–S48. 27586803

[37] Brennan FR, Kiessling A. In vitro assays supporting the safety assessment of immunomodulatory monoclonal antibodies. Toxicol In Vitro 2017;45:296–308. 28263892 10.1016/j.tiv.2017.02.025

[38] Chouinard L, Felx M, Mellal N et al. Carcinogenicity risk assessment of romosozumab: a review of scientific weight-of-evidence and findings in a rat lifetime pharmacology study. Regul Toxicol Pharmacol 2016;81:212–222. 27569204 10.1016/j.yrtph.2016.08.010

[39] Brennan FR, Cavagnaro J, McKeever K et al. Safety testing of monoclonal antibodies in non-human primates: case studies highlighting their impact on risk assessment for humans. MAbs 2018;10:1–17. 28991509 10.1080/19420862.2017.1389364 PMC5800363

[40] Bode G, Clausing P, Gervais F et al. The utility of the minipig as an animal model in regulatory toxicology. J Pharmacol Toxicol Methods 2010;62:196–220. 20685310 10.1016/j.vascn.2010.05.009

[41] Heining P, Ruysschaert T The use of Minipig in drug discovery and development: pros and cons of Minipig selection and strategies to use as a preferred nonrodent species. Toxicol Pathol 2016;44:467–473. 26674804 10.1177/0192623315610823

[42] Colleton C, Brewster D, Chester A et al. The use of Minipigs for preclinical safety assessment by the pharmaceutical industry: results of an IQ DruSafe Minipig survey. Toxicol Pathol 2016;44:458–466. 27006130 10.1177/0192623315617562

[43] Egli J, Schlothauer T, Spick C et al. The binding of human IgG to Minipig FcγRs—implications for preclinical assessment of therapeutic antibodies. Pharm Res 2019:36:article 47. 10.1007/s11095-019-2574-y PMC6373530 30721414

[44] Ennis M, Lorenz W, Kapp B et al. Comparison of the histamine-releasing activity of cremophor E1 and some of its derivatives in two experimental models: the in vivo anaesthetized dog and in vitro rat peritoneal mast cells. Agents Actions 1985;16:265–8. 2409775 10.1007/BF01983156

[45] Eschalier A, Lavarenne J, Burtin C et al. Study of histamine release induced by acute administration of antitumor agents in dogs. Cancer Chemother Pharmacol 1988;21:246–50. 2452032 10.1007/BF00262779

[46] English V. Immunocore: a regulatory first? MedNous. 2011;2011:3–15.

[47] Ryan P, Hammond S, Ren S et al. In Vitro MABEL approach for nonclinical safety assessment of MEDI-565 (MT111) In: Altex Proceedings, 8th World Congress on Alternatives and Animal Use in the Life Sciences. 2011, 85–87.

